# Sexual size dimorphism is not associated with the evolution of parental care in frogs

**DOI:** 10.1002/ece3.1263

**Published:** 2014-10-01

**Authors:** Melanie J Monroe, Suzanne H Alonzo

**Affiliations:** Department of Ecology and Evolutionary Biology, Yale UniversityP.O. Box 208106, New Haven, 06520-8106, Connecticut

**Keywords:** Amphibians, comparative methods, macroevolution, phylogeny, sexual selection

## Abstract

Sex differences in parental care are thought to arise from differential selection on the sexes. Sexual dimorphism, including sexual size dimorphism (SSD), is often used as a proxy for sexual selection on males. Some studies have found an association between male-biased SSD (i.e., males larger than females) and the loss of paternal care. While the relationship between sexual selection on males and parental care evolution has been studied extensively, the relationship between female-biased SSD (i.e., females larger than males) and the evolution of parental care has received very little attention. Thus, we have little knowledge of whether female-biased SSD coevolves with parental care. In species displaying female-biased SSD, we might expect dimorphism to be associated with the evolution of paternal care or perhaps the loss of maternal care. Here, drawing on data for 99 extant frog species, we use comparative methods to evaluate how parental care and female-biased SSD have evolved over time. Generally, we find no significant correlation between the evolution of parental care and female-biased SSD in frogs. This suggests that differential selection on body size between the sexes is unlikely to have driven the evolution of parental care in these clades and questions whether we should expect sexual dimorphism to exhibit a general relationship with the evolution of sex differences in parental care.

## Introduction

Female parental care is generally more common than male care (Darwin 1874; Bateman 1948; Trivers 1972; Clutton-Brock 1991; Queller 1997; Kokko and Jennions 2003, 2008). The most common explanation for this predominance of female parental care is that the magnitude and direction of sexual selection on the sexes differs between males and females. Because females typically invest more per gamete than do males, female fitness is argued to be limited by the number of gametes produced while male fitness is limited by access to females (Bateman 1948). In many species, particularly those with external fertilization, the ability of females to produce gametes is mainly limited by body size and the ability to obtain enough resources to sustain or increase gamete production (Shine 1988; Honĕk 1993). In species where females are larger than males, it is often assumed that fecundity selection (i.e., natural selection acting in favor of larger clutch or egg sizes) on females is stronger than sexual selection on male size (Shine 1979; Andersson 1994). In contrast, in species where males are larger than females, it is typically assumed that sexual selection favoring larger males is stronger than selection on female fecundity and body size (Darwin 1874; Trivers 1972; Andersson 1994). Sexual size dimorphism may therefore represent the balance between fecundity selection on females and sexual selection on males (Fairbairn 1997, 2013). Females may experience a fitness trade-off between providing parental care and acquiring energy for egg production, such that strong selection on female fecundity could disfavor maternal care (Gross and Sargent 1985). Similarly, strong sexual selection on males is typically argued to disfavor the evolution of paternal care (e.g., Clutton-Brock 1991; Queller 1997; Kokko and Jennions 2008). Thus, parental care theory predicts an association between the presence and direction of sexual size dimorphism (SSD) and which, if any, sex provides care (Maynard Smith 1977; Houston and McNamara 2002; Kokko and Jennions 2008).

A number of studies on birds, fishes, mammals, and reptiles show clear patterns between male-biased SSD and female parental care (i.e., if males are larger than females, females will care, Clutton-Brock 1989, 1991; Gonzalez-Voyer et al. 2008). However, some comparative studies on fishes suggest that the larger of the sexes, often males, will provide care if providing care allows them increased mating opportunities (Ah-King et al. 2005; Mank et al. 2005). On the other hand, comparative studies on birds suggest that the smaller parent will typically care, regardless of the parent's sex, due to the energetic costs and limitations of the larger parent (Jönsson and Alerstam 1990; Reynolds and Székely 1997). If the presence and direction of SSD indicates the balance between sexual selection on males and fecundity selection on females, we might expect the evolution of female-biased SSD to be associated with paternal care and/or the loss of maternal care. Although some of the above studies have generally investigated the relationship between SSD and whether males or females provide parental care (e.g., Jönsson and Alerstam 1990; Reynolds and Székely 1997; Han and Fu 2013), relatively few, if any, investigators have specifically tested how female-biased SSD affects the evolution of parental care or the specific type of parental care provided (e.g., biparental, maternal, and paternal).

As described above, large female body size is often thought to evolve through natural selection favoring an increase in fecundity, either in the form of increased number of eggs (i.e., larger clutch sizes), or increased egg size (e.g., Jönsson and Alerstam 1990; Kolm et al. 2007; Gomez-Mestre et al. 2012; Han and Fu 2013). In contrast, studies in frogs have suggested that female-biased SSD results, not from an increase in female size, but from a decrease in male size (Trivers 1972; Monnet and Cherry 2002). This may be due to higher male mortality resulting in earlier male maturation, thus smaller body size at sexual maturity (Monnet and Cherry 2002). This makes it unclear what relationship between sexual size dimorphism and parental care is expected in frogs. A recent study found that high female-biased SSD (males and females more different from one another) was associated with larger clutch sizes, whereas lower female-biased SSD (males and females more similar to one another) was associated with smaller clutch sizes and parental care, that is, relaxed selection on female fecundity. This may suggest that a tendency toward monomorphism could be associated with parental care, particularly when males are present, in frogs (Han and Fu 2013). While this study examined whether the presence or absence of parental care was associated with SSD, it did not consider whether an association exists between SSD and the form of parental care (e.g., biparental, maternal or parental). Yet, as described above, we might expect female-biased SSD to be associated with the evolution of paternal care and the loss of maternal care.

To study how female-biased SSD is associated with parental care, we use phylogenetic comparative methods to investigate the relationships between body size differences (female body size–male body size) and the presence and absence of parental care and which parent provides care (e.g., when females are larger, do males care?). We use data on 99 frog species to conduct these investigations. The majority of all frog species display female-biased SSD (Han and Fu 2013) and several monophyletic clades display a multitude of parental care types (no care, male only care, female only care, and biparental care (Summers et al. 1999, 2006; Wells 2007; Han and Fu 2013)). Thus, frogs are an excellent group by which to investigate the coevolution of female-biased SSD and parental care.

## Methods

Our data set consists of the *Dendrobates*, commonly known as poison dart frogs, all of which provide parental care (in the form of female only, male only or biparental care (Summers et al. 1999, 2006)). The *Eleutherodactylus*, or rain frogs, display no care, male only, or female only care (Wells 2007). The, *Hyla*, or tree frogs, and the *Rana*, or true frogs, provide no or very little care to their offspring (Crump 1996). Using this data, we examine the coevolution of female-biased SSD and the presence and absence of parental care, parental care type, as well as evolutionary transitions between parental care types. We first test two hypotheses regarding the coevolution of female-biased SSD and parental care. (1) Similar to recent studies (Han and Fu 2013), we test whether or not there is an association between the presence or absence of parental care and female-biased SSD. (2) In contrast to recent studies (Han and Fu 2013), however, we examine not only whether there is an association between the presence or absence of males or females caring for offspring but also whether there is an association between the evolution of type of parental care (biparental, maternal, paternal) and female-biased SSD. We use a method of phylogenetic generalized least squares and a data set of 72 species to test these two hypotheses. Additionally, this data set allows us to consider some long standing arguments regarding evolutionary transitions between male only and female only care (Zimmermann and Zimmermann 1984, 1988; Gross and Sargent 1985; Waygoldt 1987). For many ectotherms, especially amphibians, it has often been hypothesized that biparental care is a necessary stepping stone between male only and female only care [i.e., male only care → biparental care → female only care (Zimmermann and Zimmermann 1984, 1988; Waygoldt 1987 but see Summers et al. 1999, 2006)]. Here, we test this hypothesis by calculating transition rates between the different types of parental care [BayesTraits (Pagel and Meade 2006)].

### Data collection

To establish our data set, we searched for information for each species using current and synonym species names coupled with combinations of the following search terms; parental, care, investment, offspring, sex, size, difference, dimorphism, snout-to-vent length (SVL), length, male, and female. Data were obtained using search engines including Google Scholar and ISI Web of Science as well as databases such as AmphibiaWeb, IUCN redlist, and Proyecto Coquì and compiled data sets from Summers et al. (2006), Wells (2007) and Han and Fu (2013).

Parental care type for each species was classified into one of four categories based on the literature: no care (*Eleutherodactylus*, *Hyla*, *Rana*), male only care, female only care (*Eleutherodactylus*, *Dendrobates*) and biparental care (*Dendrobates*). For these care types, we were able to accumulate data for 99 species. Continuous data on body size for both males and females were available in the literature for some, but not all, species. For example, while many of the well-studied frog species have data available for both parental care and body size (e.g., *Dendrobates arboreus*, *Eleutherodactylus coqui*), others in the same clade do not (e.g., *D. amazonicus*, *E. caribe*). Likewise, covariates such as environmental and life history differences which may explain the evolution of parental care and body size differences between males and females appear to be unavailable for many species. Thus, we were able to collect body size data on both sexes for 72 species.

### Phylogenetic relationships

We extracted rooted phylogenies from Pyron and Wiens 2013 amphibian phylogeny, as this is the most complete amphibian phylogeny to date. Species for which we did not have data were pruned from the phylogeny.

### Evolutionary correlations between female-biased SSD and parental care

To determine whether or not differential selection on the sexes is associated with the evolution of parental care type (presence/absence, male/female only, and biparental), we ran a series of phylogenetic generalized least square model (PGLS) evaluating body size differences (log_10_(female body size(mm)) − log_10_(male body size(mm))) (dependent variable) against parental care types (explanatory variable).

A PGLS is often perceived as an extension of an ordinary least squares method, used for estimating the unknown parameters in a linear regression model. However, in a PGLS, the assumptions that (1) the data have the same variance, and (2) covariances are equal to zero, are relaxed. Phylogenetic trees are included as covariance matrices, and polytomies can be incorporated into the analyses (Pagel 1999a; Freckleton et al. 2002).

For our PGLS analyses, we ran analyses on the presence or absence of parental care, each type of care as one variable (i.e., care types were coded as 0 = no care, 1 = male only care, 2 = female only care, and 3 = biparental care) and individually (i.e., as the presence or absence of one care type) against body size differences (log_10_(female body size) − log_10_(male body size)).

### Transitions between types of parental care

To determine how parental care transitions from one type to another (e.g., male only → female only), we used the MULTISTATE module from BayesTraits (Pagel 1994, 1999b; Pagel and Meade 2006). MULTISTATE uses Bayesian methods to determine which character state is more likely (ancestral to descendent transitions) given the phylogenetic relationships between species and the extant character state data (i.e., tip states). During this process, MULTISTATE estimates the probability of rate changes between states. For example, MULTISTATE can estimate the rate, log-likelihood and harmonic mean that a species that provides male only care has transitioned/evolved from a species that did not provide parental care. Similarly, MULTISTATE estimates the reverse transition that a species that does not provide parental care has evolved from a species that provided male only care. Transition rates, log-likelihood ratios, and harmonic means are originally calculated under the assumption that different states are able to transition in a completely unconstrained manner. To test whether or not the differences in transition rates for each state are significant, the user runs a second analysis using the same parameters as in the first analysis (in our case, we ran a Monte Carlo Markov Chain (MCMC) with a burnin of 50,000 for 5,000,000 iterations, using an uniform prior between 0 and 100 (as these values coincided well with the maximum-likelihood tests carried out prior to the MCMC analyses) with an acceptance rate of 0.02) but constrains the rates of transition. By constraining the transition rates, MULTISTATE assumes that the transition rates between the constrained states are equal (e.g., male only→female only care=female only→male only care). Constrained rates were calculated for both reciprocal parental care transitions, for example, no care↔male only care, and on possible all transitions within a clade, for example, no care↔male only care=no care↔female only care=male only care↔female only care. This was carried out to determine not only whether unconstrained rates of transition were more informative than constrained rates between reciprocal transitions, but to determine whether in those transitions where constrained rates were equally as informative as unconstrained rates, the reciprocal constrained transitions were more informative than if all rates of transition for all parental care types were equal. To determine how informative unconstrained rates were, we calculated Bayes factors for each transition (reciprocal and all transitions) using the highest harmonic mean from each of our constrained and unconstrained analyses (׀unconstrained harmonic mean׀ – ׀constrained harmonic mean׀). If the Bayes factor was greater than two, positive support for the unconstrained analysis would be assumed. We ran this analysis for all possible parental care transitions: no care ↔ male only care, no care ↔ female only care (*Eleutherodactylus*), female only care ↔ male only care (*Eleutherodactylus*, *Dendrobates*), male only care ↔ biparental care, and female only care ↔ biparental care (*Dendrobates*).

## Results

### Differences between female-biased SSD and the presence or absence of parental care

**Figure 1:**
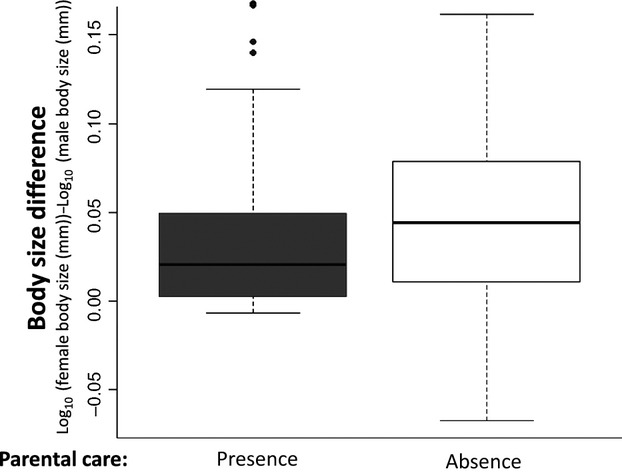
Differences in body size (log_10_(female body size (mm))-log_10_(male body size(mm))) between species that provide parental care (presence) and species that do not (absence). On average it seems that males and female of species that provide care are slightly more similar to one another (mean difference = 2.35 mm) than species that do not (mean difference = 4.50). However, this difference between species that provide care and those that do not is negligible (two-sided Welch *t*-test: *t* = −0.55, DF = 66.14, *P*-value = 0.58).

To determine whether or not there is a difference in body size and female-biased SSD between species that provide parental care and those that do not, we ran a PGLS on the presence or absence of parental care using a phylogeny containing approximately 40 species that do not provide parental care and 32 that do. There is no significant correlation between the evolution of female-biased SSD and the presence or absence of parental care (Table1, Fig.1).

**Table 1:** PGLS of body size difference (log_10_(female body size (mm)) − log_10_(male body size (mm))), (dependent variable) on parental care (explanatory variable). Analyses were run to determine the effect, or lack thereof, of SSD on the presence or absence of parental care, the type of care provided and/or whether or not there was a difference in SSD depending on which sex provides care. All analyses have 72 degrees of freedom and 70 residual degrees of freedom. *λ* was estimated using maximum-likelihood methods and can be used to determine how important the topology of the phylogeny is with regards to the variables tested (1 = very important, 0 = not important at all).

Care type	*λ*	Corr.	AIC	Log-likelihood	Standard error	*t*-value	*P*-value
Care present	0.86	−0.19	−215.74	110.87	0.04	0.55	0.58
No care, ♀, ♂, ♀ + ♂	0.87	−0.06	−212.58	109.29	0.01	0.34	0.73
♀ only	0.82	−0.01	−214.07	110.03	0.02	0.53	0.60
♀ presence	0.86	−0.02	−214.03	110.02	0.02	0.48	0.64
♂ only	0.87	−0.06	−213.72	109.86	0.02	−0.22	0.83
♂ presence	0.86	−0.07	−213.71	109.86	0.02	−0.26	0.79
♀ + ♂	0.86	−0.01	−213.55	109.78	0.02	−0.05	0.96

Corr. = correlation between body size difference and parental care type, a *P*-value ≤0.05 would suggest that the correlation between the two variables is significant. None of the analyses suggest a significant correlation between body size difference (SSD) and parental care.

**Figure 2:**
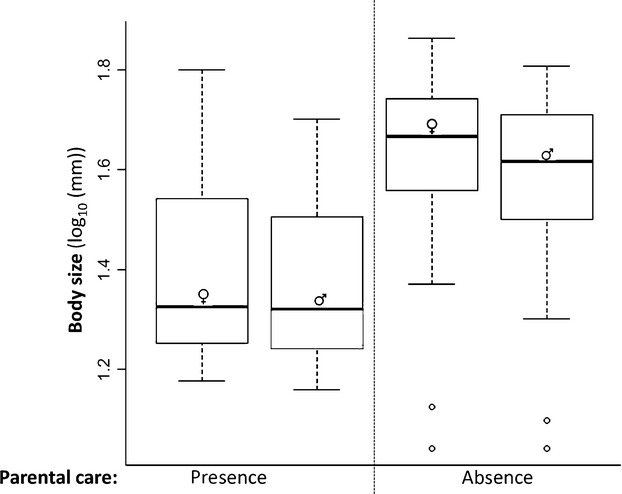
Log_10_ body size of females and males of species that do (presence) and do not (absence) provide parental care. Females and males that provide care to their offspring seem to be smaller than those males and females of species that do not provide care. Both males and females that provide care are significantly smaller than females and males that do not provide care (ANOVA: DF = 1, *F*-value = 61.99, *P*-value ≤0.001). Male and female symbols are marked directly on boxplots to represent male or female body size.

Using an ANOVA, we tested to determine whether there were differences in body size (log_10_(mm)) between males and females that do and do not provide care (e.g., the presence of care (males or females) vs. the absence of care (males or females)). We found that the presence and absence of parental care has a significant effect on body size (DF = 1, *F*-value = 61.99, *P*-value ≤ 0.001), but sex did not (DF = 1, *F*-value = 2.05, *P*-value = 0.16). Both males and females of species that provide care appear to be smaller than both males and females of species that do not provide care (Fig.2). Therefore, we find no evidence of sexual size dimorphism differing between species with and without parental care, but we do find that absolute body size of species that provide care is significantly smaller than those that do not.

### Evolutionary correlations between female-biased SSD and parental care

We used PGLS analyses to determine whether or not female-biased SSD is associated with a specific type of parental care (no care, male only, female only, and biparental). None of our analyses suggest evolutionary correlations between female-biased SSD and the type of parental care provided (Table1).

### Transitions between types of parental care

To determine the rates of transition between different types of parental care, we used the Bayesian method of MULTISTATE in BayesTraits (Pagel 1999b; Pagel and Meade 2006). According to the Bayes factors, none of the unconstrained rates of transition were significantly different than the reciprocal constrained rates of transition (Table2). Likewise, when all rates are constrained in the *Eleutherodactylus*, that is, no care↔male only care = no care↔female only care = male only care↔female only care = 1.14, constrained rates are equally informative as unconstrained (Bayes Factor = 0.94). However, when all rates are constrained in the Dendrobates, male only care↔female only care = male only care↔biparental care = female only care↔biparental care = 7.58, unconstrained (Bayes factor = 2.83) and reciprocal constrained analyses (Bayes factors: male only↔female only care = 2.72, male only↔biparental care = 2.69, and female only↔biparental care = 2.83) suggest that when all rates are constrained, results are significantly worse than whether unconstrained or constrained reciprocally.

**Table 2:** Rates of transition between types of parental care. Transition rates were calculated using the MULTISTATE module in Bayes Traits (Pagel 1994, 1999b; Pagel and Meade 2006). Both unconstrained (U) and constrained (C) analyses were run to determine whether or not unconstrained rates were more likely than constrained rates. None of the Bayes Factors (BF), calculated using the harmonic means from unconstrained and constrained analyses, suggest that unconstrained transition rates are more likely than constrained rates (i.e., BF < 2.0).

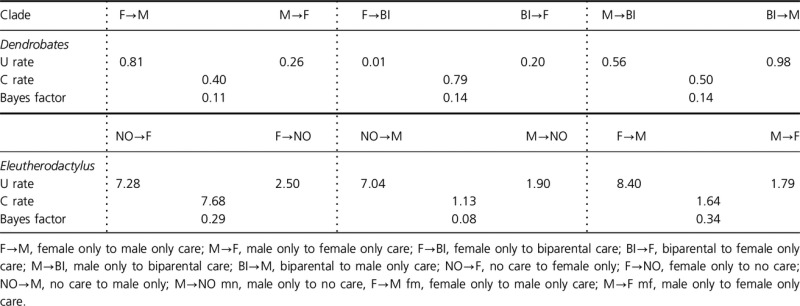

## Discussion

Previous comparative studies have found associations between male-biased SSD and the loss of paternal care in a variety of taxa. Similarly, studies of shorebirds comparing species with both female-biased and male-biased SSD have demonstrated that the smaller sex is more likely to provide care (Jönsson and Alerstam 1990; Reynolds and Székely 1997). Here, we asked whether female-biased SSD is similarly associated with the loss of maternal care in groups where female-biased SSD is common. We find no general evidence that female-biased SSD is associated with the evolution of parental care. Nor do we find evidence that female-biased SSD is associated with the loss of maternal care. However, both males and females that provide parental care are smaller than species that do not provide care. Thus, we find that absolute (total body size) rather than relative body size (SSD) coevolves with parental care in these clades.

### The evolution of female-biased SSD and parental care

Using a data set of 99 extant frog species, we find no general evidence that female-biased SSD or lack thereof is associated with the presence or absence of parental care. In contrast, a recent study using 130 frog species distributed across the complete Pyron and Weins (2011) frog phylogeny suggested that a decrease in female-biased SSD toward monomorphism or male-biased SSD is associated with the evolution of parental care (Han and Fu 2013). Our results, based on slightly fewer species overall, but more species within each clade do not support this claim. One possible explanation for this difference between the two studies is that the relationship between SSD and care may exist in some clades (e.g., those not included in our study) and may be absent in others (e.g., those included in our study). A second possibility is that extreme female-biased SSD in some species may skew the data such that clades without a similarly strong female-biased SSD may appear to evolve toward monomorphism (and parental care). Our study finds that species providing care are smaller than species that do not provide care. However, the difference in body size between males and females that provide care was not significantly smaller than species that do not provide care. Parental care and SSD data on a greater number of frog species will be necessary to resolve more fully whether and what kind of a relationship exists between the evolution of parental care and sexual size dimorphism.

### Transitions between types of parental care

No parental care is the likely ancestral character state for all frogs (Waygoldt 1987; Crump 1996). However, parental care has evolved in many frog clades (Wells 2007) and is hypothesized to evolve in correlation with many different ecological factors. For example, the evolution of parental care in frogs is associated with a decrease in pond size, an increase in the number or type of predators (Shine 1989; Brown et al. 2008, 2010), terrestrial or stream breeding (Waygoldt 1987; Gomez-Mestre et al. 2012), and following an increase in egg size (Summers et al. 2006). Which type of care evolves directly from no care and why that type of care evolves is somewhat uncertain. However, it is often suggested that the mode of fertilization, that is, internal versus external, may play a role in which sex cares: those species displaying internal fertilization will develop female care and those displaying external fertilization will develop male care (Trivers 1972). Other hypotheses, however, such as future reproductive success have been suggested to determine which sex will provide care. It is assumed that females are limited by the number of gametes they are able to produce, while males are limited by number of mates (Bateman 1948; Shine 1988; Honĕk 1993). Thus, under the assumption that parental care is essential for adult fitness, if food, for example, energy for gamete production, is plentiful, females should care, whereas if mating opportunities are frequent, males should care (Crump 1996). Our results suggest that the evolution from no care to female care is just as likely as the evolution of male care from no care, based on our constrained (all states constrained) and unconstrained rates of transition.

Once care has evolved, however, transitions between care types often occur (Gross and Sargent 1985; Summers et al. 1999, 2006; Wells 2007; Klug et al. 2013). Recent theory suggests that transitions from one care type to another will occur when differences between male and female life history traits increase (Klug et al. 2013). In particular, rates of sex specific maturation and/or mortality (eggs, juveniles, adults) influence which type of parental care transition will occur. For example, Klug et al. (2013) predicts that slow egg maturation and higher mortality rates in males will result in male care because males will have a higher fitness gain from caring for current offspring than they will if they are unable to reproduce again. Likewise, if females show slow egg maturation and high mortality rates, they would be more likely to invest more in parental care of offspring than would males. Again, our results suggest that transitions between types of parental care are equally likely (reciprocal constrained rates=unconstrained rates), regardless of the ancestral care type. However, of the frog species that provide care, there seem to be many more species of frogs (in our data set) that provide male only care (58%) as opposed to female only (28%) or biparental care (13%). Thus, according to the theory proposed by Klug et al. (2013), because male care seems to evolve more often than female or biparental care future studies, conducted once additional data becomes available, may predict that males exhibit higher mortality rates and slower rates of egg development than females in many of the *Dendrobates* and *Eleutherodactylus* species.

## Conclusion

Our study uses comparative methods to evaluate rates of transition between parental care types and whether or not differential selection on the sexes (in the form of SSD) is associated with the evolution of parental care. Despite rates of transition between parental care types being equally informative when constrained as when unconstrained, we find no evidence in support of the hypothesis that biparental care is stepping stone between male and female only care (Zimmermann and Zimmermann 1984, 1988; Waygoldt 1987). In fact, because no *Eleutherodactylus* species, of which we are aware, display biparental care, yet many species display male only care or female only care, this alone suggests that biparental care may not generally be an intermediate step between two types of care (Summers et al. 1999, 2006). Furthermore, we find no evidence that female-biased SSD is associated with the loss of maternal care. Further evaluation of why female-biased SSD occurs may provide some insight into why female-biased SSD is generally not correlated with parental care. For example, size differences between the sexes could also be due to selection on age at maturation (large females may simply be older than males (Monnet and Cherry 2002)) and not a result of strong selection on female fecundity (Andersson 1994; Han and Fu 2013). This raises the question of whether male-biased SSD is fundamentally different from female-biased SSD or whether studies focused on male-biased SSD have given us an incomplete understanding of how differential selection on the sexes affects parental care evolution.

Generally, we find no evidence of an association between the evolution of female-biased SSD and the presence or absence of parental care in these frogs. It is unclear whether this lack of correlation is because female-biased SSD represents an increase in female fecundity, or because it represents a decrease in male body size, these two traits (SSD and parental care) do not appear to be experiencing correlated selection. Thus, although there is a difference in absolute body size between species that do and do not provide care, we do not find support for a general relationship between SSD (i.e., relative body size differences) and the evolution of sex differences in parental care.

## References

[b1] Ah-King M, Kvarnemo C, Tullberg BS (2005). The influence of territoriality and mating system on the evolution of male care: a phylogenetic study on fish. J. Evol. Biol.

[b2] Andersson M (1994). Sexual selection.

[b3] Bateman AJ (1948). Intra-sexual selection in Drosophila. Heredity.

[b4] Brown JL, Morales V, Summers K (2008). Divergence in parental care, habitat selection and larval life history between two species of Peruvian poison frogs: an experimental analysis. J. Evol. Biol.

[b5] Brown JL, Morales V, Summers K (2010). A key ecological trait drove the evolution of biparental care and monogamy in an amphibian. Am. Nat.

[b6] Clutton-Brock TH (1989). Mammalian mating systems. Proc. R. Soc. B Biol. Sci.

[b7] Clutton-Brock TH (1991). The evolution of parental care.

[b8] Crump ML (1996). Parental care among the amphibia. Adv. Study Behav.

[b9] Darwin C (1874). The descent of man, and selection in relation to sex.

[b10] Fairbairn DJ (1997). Allometry for sexual size dimorphism: pattern and process in the coevolution of body size and males and females. Annu. Rev. Ecol. Syst.

[b11] Fairbairn DJ (2013). Odd couples: extraordinary differences between the sexes in the animal kingdom.

[b12] Freckleton RP, Harvey PH, Pagel M (2002). Phylogenetic analysis and comparative data: a test and review of evidence. Am. Nat.

[b13] Gomez-Mestre I, Pyron RA, Wiens JJ (2012). Phylogenetic analyses reveal unexpected patterns in the evolution of reproductive modes in frogs. Evolution.

[b14] Gonzalez-Voyer A, Fitzpatrick JL, Kolm N (2008). Sexual selelction determines parental care patterns in cichlid fishes. Evolution.

[b15] Gross MR, Sargent RC (1985). The evolution of male and female parental care in fishes. Am. Zool.

[b16] Han X, Fu J (2013). Does life history shape sexual size dimorphism in anurans? A comparative analysis. BMC Evol. Biol.

[b17] Honĕk A (1993). Intraspecific variation in body size and fecundity in insects: a general relationship. Oikos.

[b18] Houston AI, McNamara JM (2002). A self-consistent approach to paternity and parental effort. Philos. Trans. R Soc. B Biol. Sci.

[b19] Jönsson PE, Alerstam T (1990). The adaptive significance of parental role division and sexual size dimorphism in breeding shorebirds. Biol. J. Linn. Soc.

[b20] Klug H, Bonsall MB, Alonzo SH (2013). Sex differences in life history drive evolutionary transitions among maternal, paternal, and bi-parental care. Ecol. Evol.

[b21] Kokko H, Jennions M (2003). It takes two to tango. Trends Ecol. Evol.

[b22] Kokko H, Jennions MD (2008). Parental investment, sexual selection, and sex ratios. J. Evol. Biol.

[b23] Kolm N, Stein RW, Mooers AØ, Verspoor JJ, Cunningham EJA (2007). Can sexual selection drive female life histories? a comparative study on Galliform birds. Eur. Soc. Evol. Biol.

[b24] Mank JE, Promislow DEL, Avise JC (2005). Phylogenetic perspectives in the evolution of parental care in ray-finned fishes. Evolution.

[b25] Maynard Smith J (1977). Parental investment: a prospective analysis. Anim. Behav.

[b26] Monnet J-M, Cherry MI (2002). Sexual size dimorphism in anurans. Proc. R. Soc. Lond. B Biol. Sci.

[b27] Pagel M (1994). Detecting correlated evolution on phylogenies: a general method for the comparative analysis of discrete characters. Proc. R. Soc. B Biol. Sci.

[b28] Pagel M (1999a). Inferring the historical patterns of biological evolution. Nature.

[b29] Pagel M (1999b). The maximum likelihood approach to reconstructing ancestral character states of discrete characters on phylogenies. Syst. Biol.

[b30] Pagel M, Meade A (2006). Bayesian analysis of correlated evolution of discrete characters by reversible-jump Markov chain Monte Carlo. Am. Nat.

[b501] Pyron RA, Wiens JJ (2011). A large-scale phylogeny of amphibia with over 2800 species and a revised classification of extant frogs, salamanders, and caecilians. Molecular Phylogenetics and Evolution.

[b31] Pyron RA, Wiens JJ (2013). Large-scale phylogenetic analyses reveal the causes of high tropical amphibian diversity. Proc. R. Soc. B Biol. Sci.

[b32] Queller DC (1997). Why do females care more than males?. Proc. R. Soc. B Biol. Sci.

[b33] Reynolds JD, Székely T (1997). The evolution of parental care in shorebirds: life histories, ecology, and sexual selection. Behav. Ecol.

[b34] Shine R (1979). Sexual selection and sexual dimorphism in the amphibia. Copeia.

[b35] Shine R (1988). The evolution of large body size in females: a critique of Darwin's “fecundity advantage”. Am. Nat.

[b36] Summers K, Weight LA, Boag P, Bermingham E (1999). The evolution of female parental care in poison frogs of the genus Dendrobates: evidence from mitochondrial DNA sequences. Herpetologica.

[b37] Summers K, McKeon CS, Heying H (2006). The evolution of parental care and egg size: a comparative analysis in frogs. Proc. R. Soc. B Biol. Sci.

[b39] Trivers RL, Campbell B (1972). Parental investment and sexual selection. Sexual selection and the descent of man.

[b40] Waygoldt P (1987). Evolution of parental care in dart poison frogs (Amphibia: Anura: Dendrobatidae). Zeitschrift fur Zoologische Systematik und Evolutionsforschung.

[b41] Wells KD (2007). The ecology and behavior of amphibians.

[b42] Zimmermann E, Zimmermann H (1984). Durch nachzucht erhalten; Baumsteigfrosche Dendrobates quinquevittatus und D. reticulates. Aquarien Mag.

[b43] Zimmermann E, Zimmermann H (1988). Ethotaxonomie und zoographische artenggruppenbildung bei pfeilgiftfroschen (Anura: Dendrobatidae). Salamandra.

